# Approaches and outcomes of Robotic-Assisted Thoracic Surgery (RATS) for lung cancer: a narrative review

**DOI:** 10.1007/s11701-022-01512-8

**Published:** 2022-12-21

**Authors:** Giovanni Mattioni, Alessandro Palleschi, Paolo Mendogni, Davide Tosi

**Affiliations:** 1 Thoracic Surgery and Lung Transplantation Unit, IRCCS Foundation Ca’ Granda Ospedale Maggiore Policlinico, 20122 Milan, Italy; 2 School of Thoracic Surgery, University of Milan, 20122 Milan, Italy

**Keywords:** Robotic-Assisted Thoracic Surgery, Approaches, Lung cancer, Operative outcomes, Oncological outcomes, Lobectomy

## Abstract

Robotic-Assisted Thoracic Surgery (RATS) is considered one of the main issues of present thoracic surgery. RATS is a minimally invasive surgical technique allowing enhanced view, accurate and complex movements, and high ergonomics for the surgeon. Despite these advantages, its application in lung procedures has been limited, mainly by its costs. Since now many different approaches have been proposed and the experience in RATS for lungs ranges from wedge resection to pneumonectomy and is mainly related to lung cancer. The present narrative review explores main approaches and outcomes of RATS lobectomy for lung cancer. A non-systematic review of literature was conducted using the PubMed search engine. An overview of lung robotic surgery is given, and main approaches of robotic lobectomy for lung cancer are exposed. Initial experiences of biportal and uniportal RATS are also described. So far, retrospective analysis reported satisfactory robotic operative outcomes, and comparison with VATS might suggest a more accurate lymphadenectomy. Some Authors might even suggest better perioperative outcomes too. From an oncological standpoint, no definitive prospective study has yet been published but several retrospective analyses report oncological outcomes comparable to those of VATS and open surgery. Literature suggests that RATS for lung procedures is safe and effective and should be considered as a valid additional surgical option.

## Introduction

The employment of robotic assistance in human lives as well as in the medical field is gradually growing. Robotic systems have been introduced in surgery as an extension of the surgeon’s hands to improve procedures. One of the main objectives was also to offer the possibility of remote-control surgery. The first and probably the most common robotic surgical system is the da Vinci (Sunnyvale, California, USA), produced by Intuitive. Xi is their latest product. Since its FDA approval in 2000 the da Vinci surgical system has been spreading in surgical specialties and procedures, especially in urology [1]. In the last 10 years, even thoracic surgery has seen an increase in the use of robotic surgical systems and now Robotic-Assisted Thoracic Surgery (RATS) is considered one of its main issues. Other robotic platforms are now available and in use for thoracic surgery, as the Versius system (Cambridge, UK), produced by CMR. However, at present, RATS evidence in literature concerns only the da Vinci system. Despite RATS practical advantages, its wide introduction into routine has been slowed by costs. Even so, many thoracic teams welcomed with enthusiasm this technology, highlighting achieved results in the literature. Some surgeons reported a higher interest in RATS use in the thymic region rather than in the lung region, where benefits were believed to be less substantial. Here we report the main approaches to robotic lobectomy and discuss the role and results of RATS in lung cancer performed with the da Vinci surgical system.

## Materials and methods

**Fig. 1:**
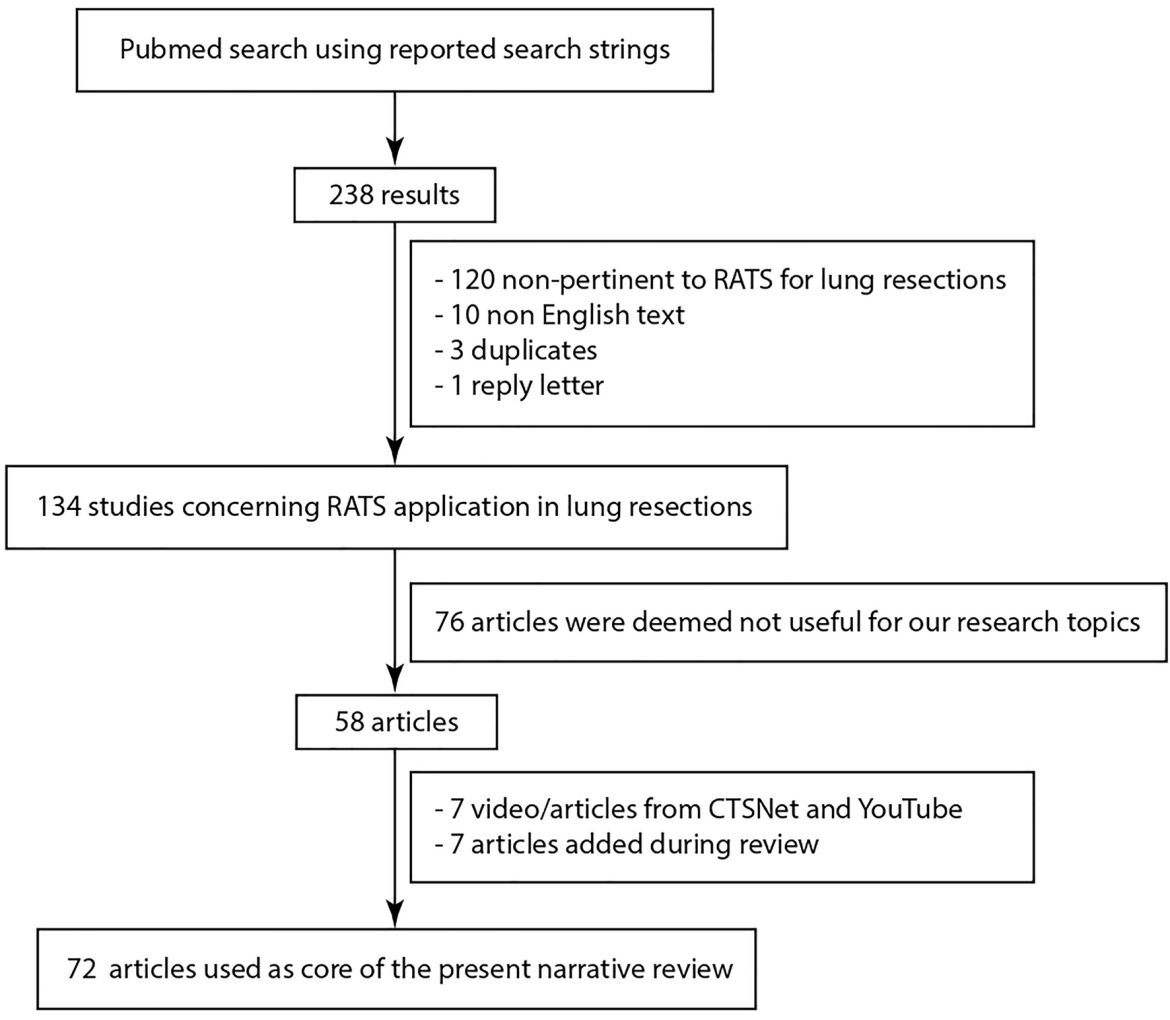
Flow diagram of articles selection from literature

The literature research was conducted using the PubMed search engine. The following search strings were used: [“robot*”[ti] AND “thoracic”[ti] AND “surgery”[ti] AND “lung”[ti]], [“robot*”[ti] AND “lung cancer”[ti] AND “lobectomy” AND “surgery”], [“trial” AND “robot*”[ti] AND (“lung cancer”[ti] OR lobectomy[ti])], [“robot*”[ti] AND (“sleeve lobectomy”[ti] OR pneumonectomy[ti] OR segmentectomy[ti]) AND “surgery”[ti]], [“robot*”[ti] AND “learning curve” AND (lobectomy[ti] OR lung[ti])]. No publication time limit was selected. Of 238 initial results, 104 articles were excluded, 120 were not related to the application of RATS to lung resection, 10 were not in English, 3 were duplicates and 1 was a reply letter. Of the 134 articles obtained, 76 were not selected because deemed not useful for our research topics: RATS approaches for lobectomy, RATS application and outcomes for lobectomy and other lung resections, RATS lymphadenectomy, RATS lobectomy learning curve. To the 58 articles identified, 7 video-articles and conference videos available on web platforms (i.e., YouTube, CTSNet) were added, and 7 more articles were added during the review process. Finally, a total of 72 articles were used as core of the present narrative review. A schematic illustration of this selection is represented in Fig. 1.

### Robotic lung surgery

Available literature extends from benign to malignant disease and comprises procedures from wedge to pneumonectomy. A limited amount of data is available for lung resections for benign diseases (i.e., bronchiectasis, intralobar sequestration, and pulmonary infections, such as tuberculosis, aspergillomas, etc.), concerning both lobectomy and segmentectomy [2–6]. In contrast, experience in lung cancer is significantly expanding and reports of benefits from RATS are emerging, as seen for robotic sleeve lobectomies. As expected, lobectomy for lung cancer is the main RATS indication for lung surgery in literature. Most of available evidence are related to this topic, which will be the focus of the present article.

Some general advantages and disadvantages of RATS lung procedures should be taken into account to help evaluate usefulness and limits.

### Pros

- Tremor filtration, more precision, and equivalence between dominant and non-dominant hands
- Increased degree of manipulative freedom
- High-definition optical system, stable camera platform, and enhanced 3D-view
- Less strain and more ergonomics
- Potential teaching superiority (with dual console) [7, 8].

Many of these aspects confirm that RATS is a technique that standardizes the surgical gesture. Differently from VATS and open surgery, the surgical movement is filtered by a robot, ensuring better movements, and the articulated wrist offers a greater range of motion. Moreover, high video quality and 3D view offer better visualization of the target. Therefore, surgical actions which may benefit from this technique are lymphadenectomy, and suturing (as during sleeve lobectomy [9] and diaphragm plication [8]).

### Cons

- Absence of haptic sensation
- Higher costs
- Prolonged operative times (especially preparation for operation)
- Difficult learning curve for the whole team [8].

So far, the main obstacle to RATS spread has been its costs. A recent systematic review highlighted that the major component of robotic costs is the disposable equipment, accounting for a difference of 1100€ per case when compared to VATS. The staff costs, due to the longer operative times, are also high [10]. An additional limit has been the absence of tactile feedback. Even if experimental systems exist, at present commercially available robotic platforms do not provide technologies that reproduce intraoperative haptic sensation [11, 12]. However, some surgeons stated that this limit can be compensated by the 3D vision and the high video quality.

### Robotic lobectomy approaches

Several robotic lobectomy approaches have been described in the literature so far. RATS approaches differ in terms of:

- Number and location of ports and/or assistant access
- Presence or absence of mini-thoracotomy
- Number of robotic arms used
- CO_2_ insufflation

**Fig. 2:**
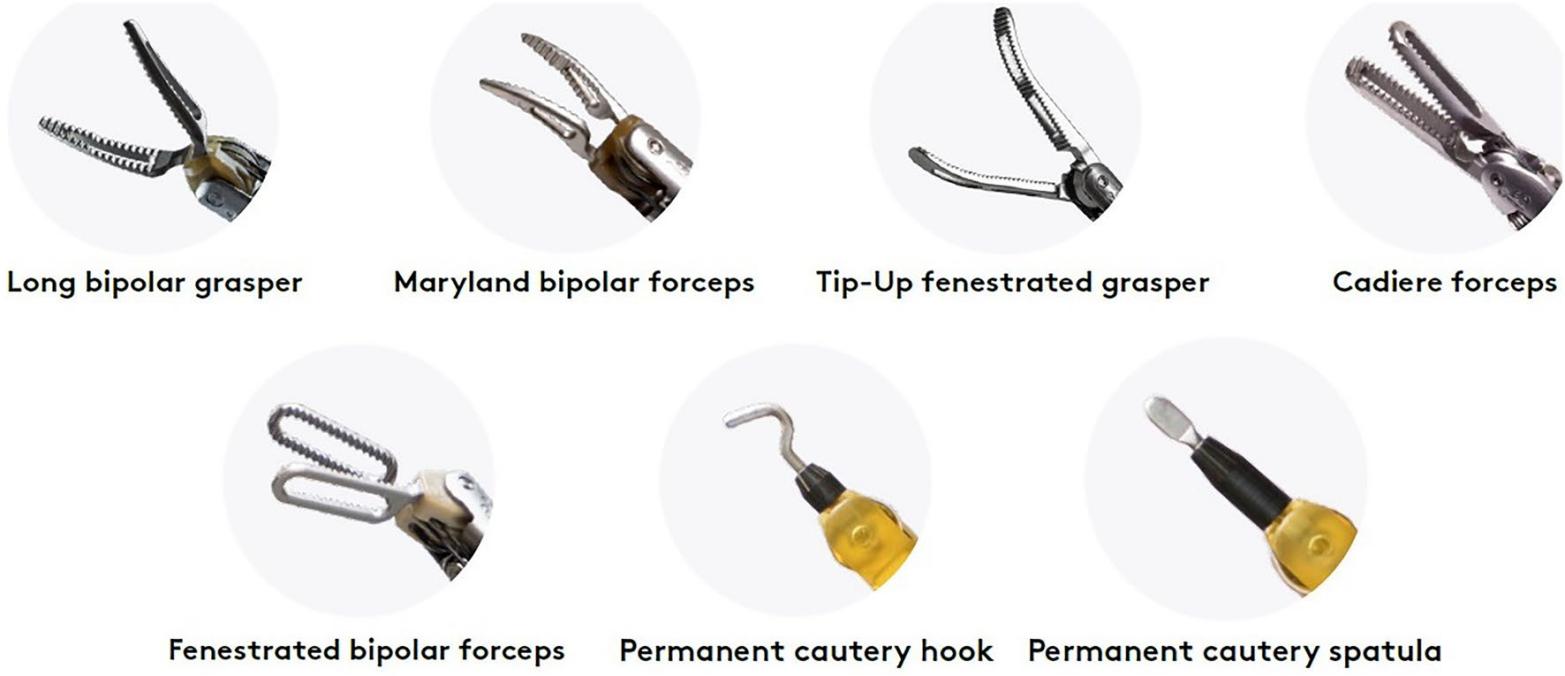
Main robotic instruments of da Vinci robotic surgical platform. Images are a gentle courtesy of Ab Medica, Italy

**Fig. 3:**
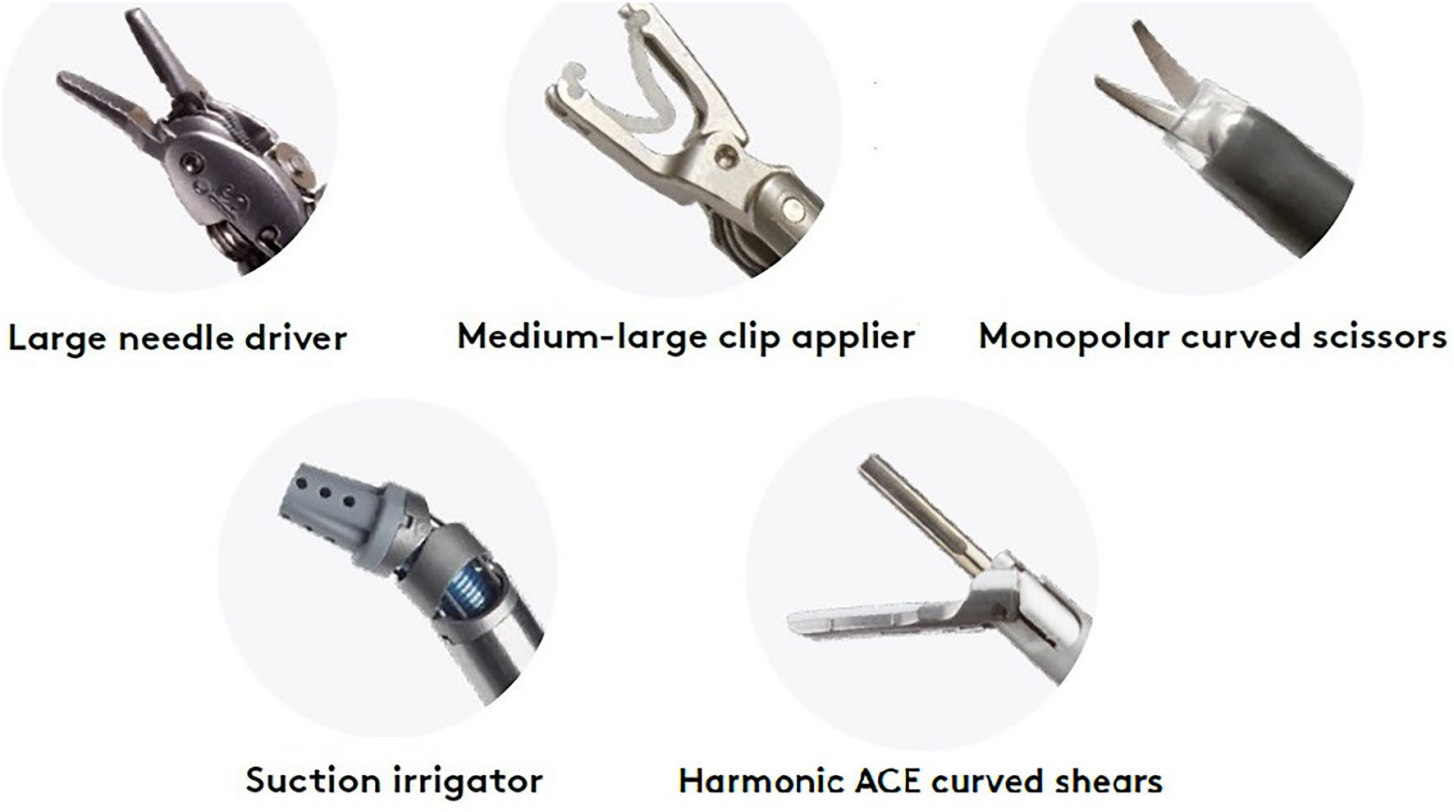
Additional robotic instruments of da Vinci robotic surgical platform. Images are a gentle courtesy of Ab Medica, Italy

Some general rules can be identified. At present, the main robotic lobectomy approaches are based on the da Vinci Si and Xi platform. Robotic ports are 8 or 12 mm in diameter and usually, the 12 mm port is used for the camera or the stapler, whereas the 8 mm one is for robotic instruments. Moreover, it is generally accepted that a minimum distance between accesses should be maintained (usually 6–9 cm). Finally, some basic robotic instruments are widely employed, such as the long bipolar grasper, the Maryland bipolar forceps, the tip-up fenestrated grasper, the Cadière forceps, the fenestrated bipolar forceps, a monopolar instrument as the permanent cautery hook, or the permanent cautery spatula (see Fig. 2). However, there are some additional available robotic instruments which are variably used, such as different types of needle drivers, clip appliers, scissors (monopolar or not), and staplers, as well as other variants of graspers and bipolar cautery, suction irrigator, and energy devices (see Fig. 3). The number of deployed robotic arms varies between 3 and 4 (one of which is dedicated to the camera). Accesses are standardized; however, most Authors recommend eventually adjusting their position based on internal anatomy, rather than just relying on external anatomy. Thus, port positioning should be performed under camera visualization.

Two main different groups of RATS approaches can be identified, one only presenting robotic ports, and another based on the presence of a mini-thoracotomy.

**Fig. 4:**
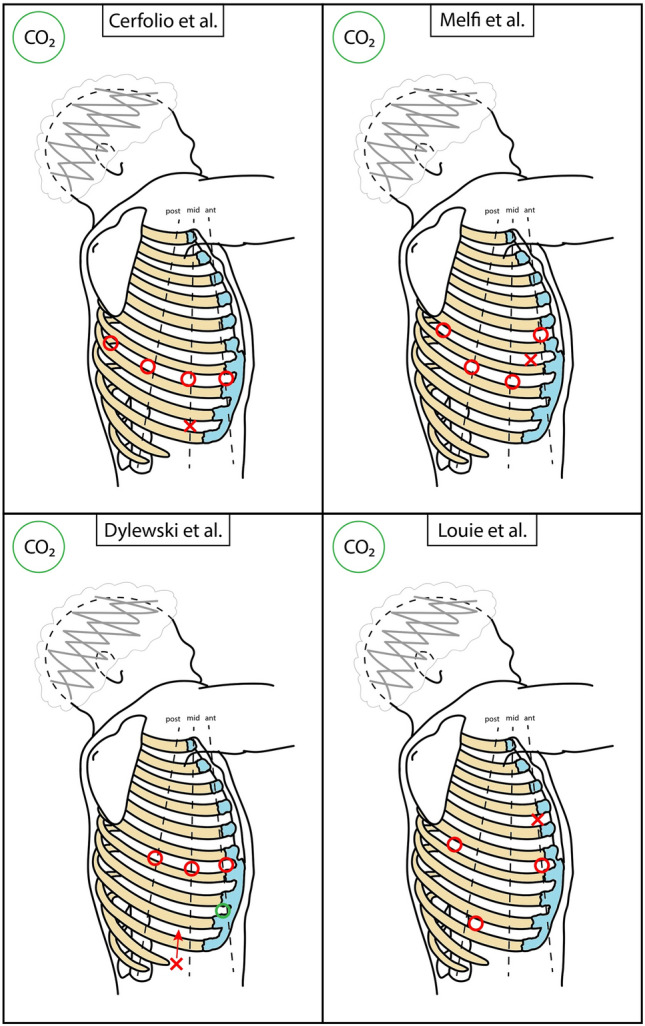
Schematic representation of main RATS accesses positioning. Dimensions and distances are not to scale, but only indicative. Red circle = robotic port; red cross = assistant access; green shape = new or alternative access (see also in the text); red arrow = tunnelling

**Fig. 5:**
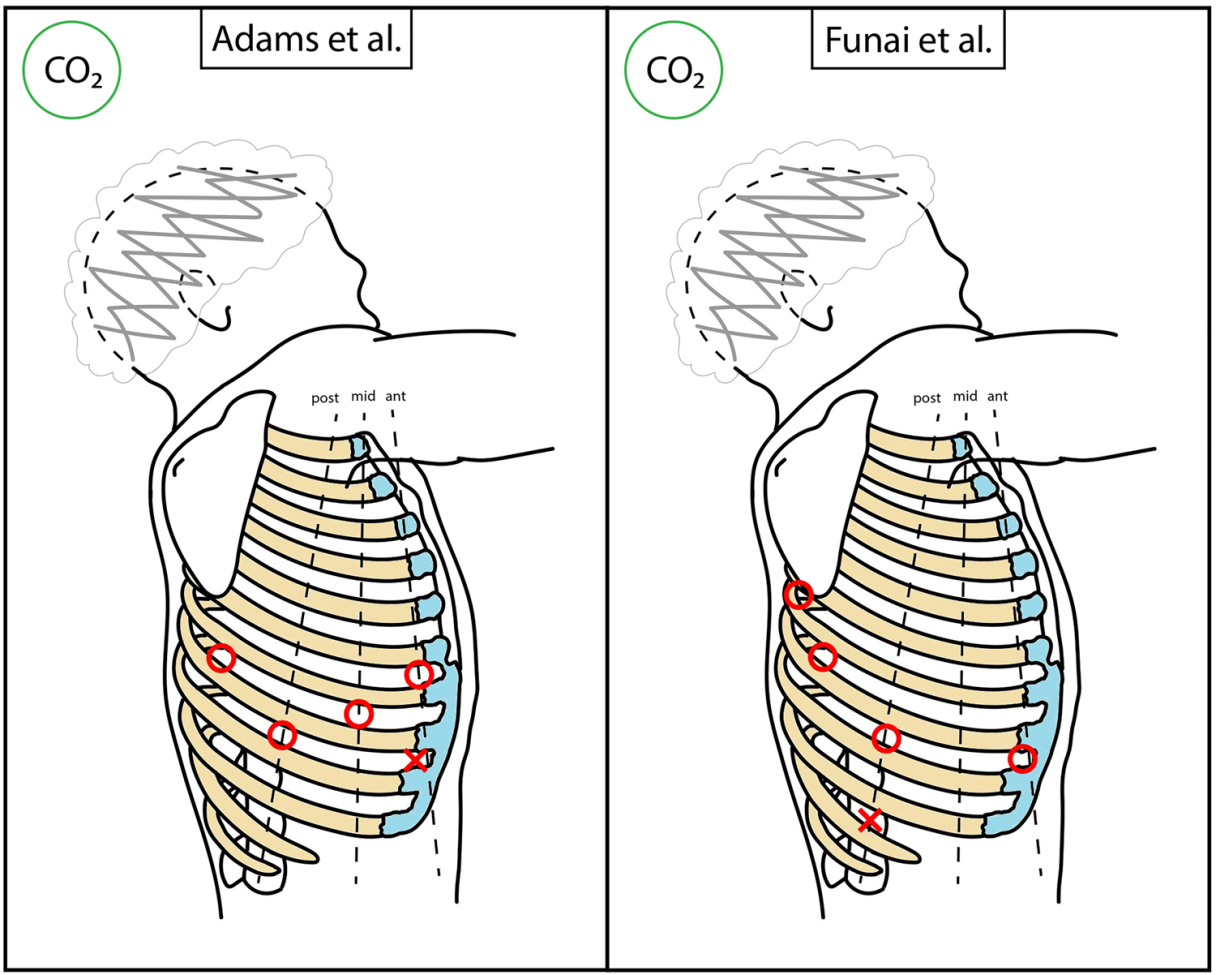
Schematic representation of main RATS accesses positioning. Dimensions and distances are not to scale, but only indicative. Red circle = robotic port; red cross = assistant access; red arrow = tunnelling

Approaches by the first group have been identified as Robotic Portal (RP) operations (termed RPL when lobectomy is performed), which only use robotic trocars ports [13]. The main literature contributors of this group are Cerfolio, Melfi, Dylewski, Louie, and Adams [14–18]. Four-arm deployment and CO_2_ insufflation are common. A schematic representation is available in Figs. 4 and 5, and in Table 1. Cerfolio’s port positioning follows one intercostal space: the 7th for upper and middle lobectomies and the 8th for lower lobectomies, on the right side [14]. One arm port is the most anterior on the anterior axillary line, another arm port is the most posterior on the paravertebral line (4 cm away from the spine processes), and the last arm port is more anterior to the latter. The camera port is more posterior to the first one. The assistant port is positioned 2–3 intercostal spaces below, forming a triangle with the first and the camera ports. CO_2_ is injected. Melfi et al. described an alternative port positioning: the camera port is placed in the 7–8th intercostal space on the midaxillary line, an arm port in the 5–6th intercostal space on the anterior axillary line, one arm port in the 6–7th intercostal space on the posterior axillary line and another one in the auscultatory area [15]. A utility port between the camera port and the anterior robotic port can be positioned for the assistant. CO_2_ is instilled at a maximum pressure of 6–8 mmHg. Dylewski et al. initially described a 3-arm approach with an assistant port placed anteriorly to the abdominal aspect of the 11th rib, from which the 10th intercostal space is reached with blunt tunnelling, a camera port above the major fissure, in the 6–7th intercostal space on the midaxillary line, and the other two arm ports are positioned in the same intercostal space, anteriorly (anterior axillary line) and posteriorly [16]. With the advent of da Vinci Xi, they moved to a 4-arm approach, adding a port in the 8–9th intercostal space, on the anterior axillary line. Here, CO_2_ is insufflated with 8 mmHg pressure [19]. Louie et al. described a 3-arm approach [17]. Two arm ports are placed in the 6th intercostal space: one on the anterior axillary line, and another inferior and posterior to the scapular tip. The camera port is placed in the 9–10th intercostal space on the posterior axillary line, while the assistant port is anteriorly in the 4th intercostal space. CO_2_ insufflation is delivered with 6 mmHg of pressure only until lung deflation. Adams et al. use a 4-arm approach, with the camera port placed on the anteroposterior midpoint of the chest, as the 7th intercostal space, midaxillary line [18]. The other two anterior and posterior arm ports are positioned directly anteriorly (as in the 6th intercostal space) and posteriorly (as in the 8th intercostal space) to the camera port, respectively. The last arm port is positioned in the 6th intercostal space, more posteriorly than the posterior arm port. An assistant port is placed above the costal margin. CO_2_ is injected with pressures ranging 8–18 mmHg. More recently, some variants of RPL have been proposed. A particular approach to right upper lobectomy was described by Funai et al. as the “Hamamatsu method” [20]. An arm port is positioned in the 8th intercostal space on the anterior axillary line, and a camera port is placed at the midpoint of the line connecting the first port and the tip of the scapula. Another arm port is in the 7th intercostal space, on the dorsal side of the lower scapula. The last arm port is in the 5–6th intercostal space, the most posterior location. A 3 cm assistant port is positioned in the 10th intercostal space, on the posterior axillary line, and presents an Alnote-Lapsingle (Alfresa; Osaka, Japan) attached to it. CO_2_ is insufflated through the port at a pressure of 8 mmHg. This approach proved helpful in visualizing more cranial structures.

**Table 1:** Main RPL approaches characteristics

Author	N° incisions	N° arms	Camera port	Assistant access	CO_2_ insufflation
Cerfolio et al. [14]	5	4	7–8th ICS, MAL	9–10th ICS, MAL	Yes
Melfi et al. [15]	4–5	4	7–8th ICS, MAL	6th ICS, MAL-AAL	Yes
Dylewski et al. [19]	5	4	6–7th ICS, MAL	10th ICS, MAL-AAL	Yes
Louie et al. [17]	4	3	9–10th ICS, PAL	4th ICS, AAL	Yes
Adams et al. [18]	5	4	7th ICS, MAL	8th ICS, AAL	Yes
Funai et al. [20]	5	4	8th ICS, PAL	10th ICS, PAL	Yes

*RPL* robotic portal lobectomy, *ICS* intercostal space, *AAL* anterior axillary line, *MAL* midaxillary line, *PAL* posterior axillary line

**Fig. 6:**
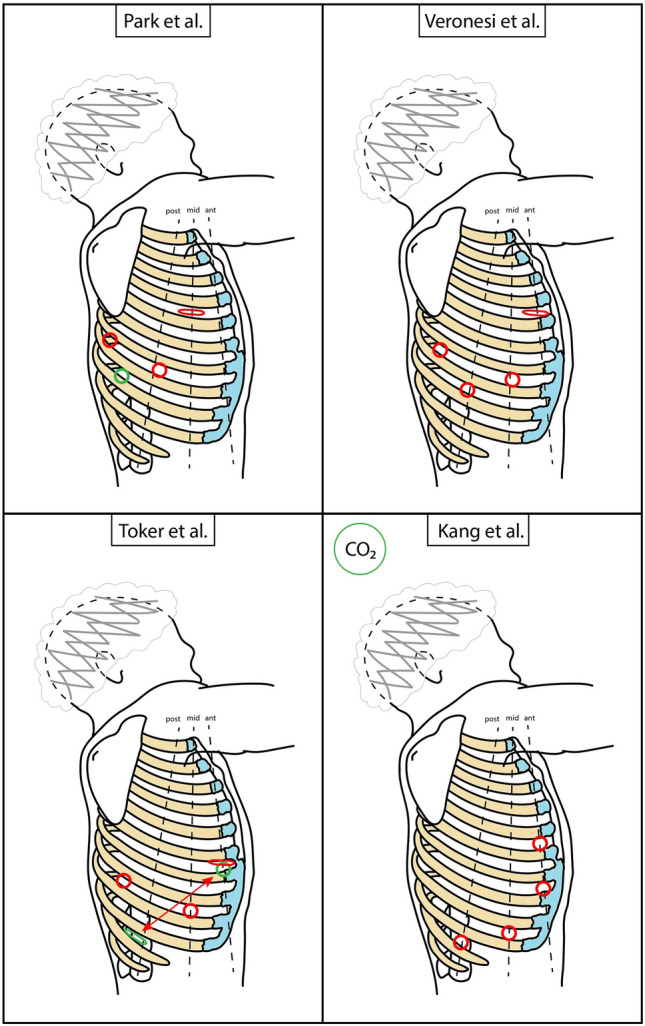
Schematic representation of main RATS accesses positioning. Dimensions and distances are not to scale, but only indicative. Red circle = robotic port; red cross = assistant access; red flattened circle = mini-thoracotomy (valid as assistant access too); green shape = new or alternative access (see also in the text); red arrow = tunnelling

The second group operations have been defined as Robotic-Assisted (RA–as before, RAL in case of lobectomy), based on the presence of a utility mini-thoracotomy [13]. Consequently, the insertion of instruments by the assistant surgeon, as well as the extraction of the specimen, can be easily performed through this access. CO_2_ insufflation is possible; however, a dedicated plastic or gel cover should be applied. The main contributors to this approach are Park, Veronesi, and Toker [21–23]. A schematic representation is available in Fig. 6 and in Table 2. Park et al. initially described a 3-arm approach, with a 3 cm mini-thoracotomy for both robotic arm port and assistant in the 4–5th intercostal space (for upper and middle-lower lobectomies, respectively) on the midaxillary line [21]. The camera port is in the 7–8th intercostal space on the posterior axillary line, and an arm port is placed just above the diaphragm posteriorly to the tip of the scapula. No CO_2_ is injected. As seen above for Dylewski, they switched to 4 arms with da Vinci Xi, adding an arm port in the 7–8th intercostal space [24]. Veronesi et al. deploy 4 arms [22]. The 3-cm utility mini-thoracotomy is performed anteriorly in the 4th intercostal space, and a robotic arm port is placed there. The other 2 arm ports are positioned in the 7th intercostal space at the tip of the scapula, and in the 8th intercostal space on the posterior axillary line. The camera port is positioned in the 7–8th intercostal space on the midaxillary line on the right, that is 2 cm posteriorly on the left (to avoid view obstacle of the heart). No CO_2_ is used. A 3-arm approach without CO_2_ is described by Toker et al. [23]. The camera port is positioned in the 8th intercostal space on the midaxillary line. The second arm port is placed posteriorly in the 8–9th intercostal space. The anterior arm port is higher in the 6–7th intercostal space. For upper lobectomies, a utility mini-thoracotomy is performed in the 10–11th intercostal space posteriorly and used only as assistant access. For lower lobectomies, the mini-thoracotomy is performed in place of the above-described anterior arm port, working as an arm port too. A 4-arm anterolateral variant of RAL is reported by Kang, with a 3 cm utility mini-thoracotomy in the 5th intercostal space on the anterior axillary line for both arm port and assistant access [25]. An arm port is in the 7th intercostal space at the anterior axillary line, the camera port is in the 9th intercostal space on the midaxillary line, and the last arm port is in the 11th intercostal space on the posterior axillary line. CO_2_ can be insufflated through a glove port. This approach is stated to avoid posterior painful accesses and facilitate a uniportal VATS approach (e.g., in case of prior wedge resection).

**Table 2:** Main RAL approaches characteristics

Author	N° incisions	N° arms	Camera port	Mini-thoracotomy*	CO_2_ insufflation
Park et al. [24]	4	4	7–8th ICS, PAL	4–5th ICS, MAL	No
Veronesi et al. [22]	4	4	7–8th ICS, MAL	4th ICS, AAL	No
Toker et al. [23]	3	3	8th ICS, MAL	10–11th ICS, PAL**	No
Kang et al. [25]	4	4	9th ICS, MAL	5th ICS, AAL	Yes

*RAL* robotic assisted lobectomy, *ICS* intercostal space, *AAL* anterior axillary line, *MAL* midaxillary line, *PAL* posterior axillary line

*Includes assistant access. **In case of inferior lobectomies: 6–7th ICS, AAL

Most active Authors on robotic thoracic surgery have been modifying and personalizing their approaches over the years according to technological advances and their growing experience; current literature testifies to this progress, as in the case of Cerfolio [14, 26], Dylewski [16, 19], and Park [21, 24].

Authors using the RP approach report some advantages: the easy use of CO_2_ that increase the operating space and reduces the probability of damaging structures, and the avoidance of tissue contact with the cold and dry room air [27]. In contrast, RA approach authors describe the possibility of lung palpation and the easy conversion to VATS [23]. As far as the number of deployed arms is concerned, using 3 arms may ideally reduce costs given the absence of 1 operating arm and relative instruments and minimize conflicts outside the body [23]. However, it should be highlighted that adding an arm could scale down the need for the assistant’s help (thus increasing the operating surgeon freedom and independence) and the number of times an instrument must be changed by the assistant [22]. However, no comparative studies have been published to date.

**Fig. 7:**
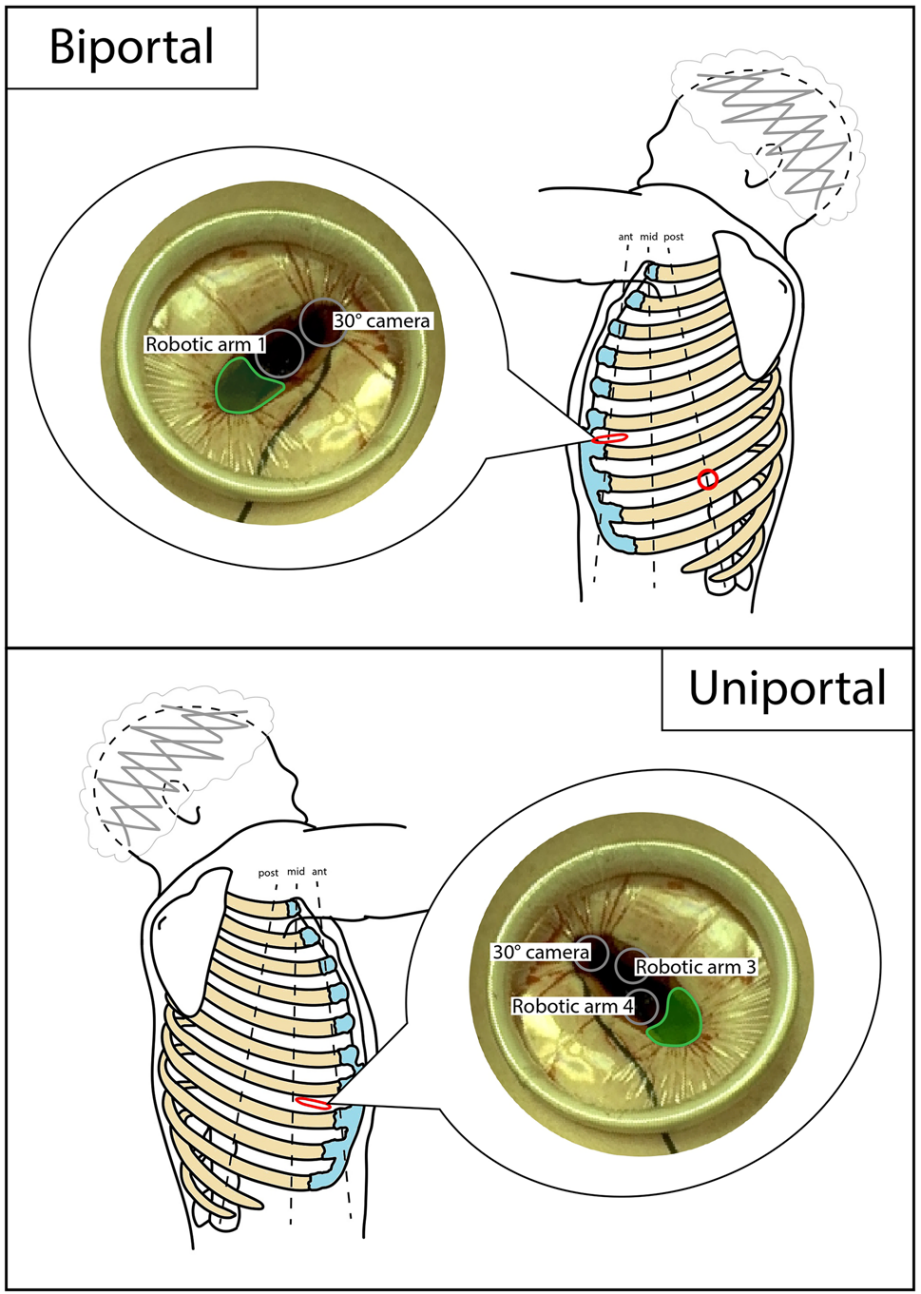
Schematic representation of biportal and uniportal RATS accesses positioning. Dimensions and distances are not to scale, but only indicative. Red circle = robotic port; red flattened circle = mini-thoracotomy (valid as assistant access too); grey circle = robotic trocar; green area = assistant area/access

In the last few years, reports on experiences of biportal and uniportal robotic approaches have been appearing in literature. Yang et al. reported a 3-arm biportal approach with an arm port in the 8th intercostal space on the posterior axillary line and 2 arms (camera included) and assistant access in a 4 cm incision in the 6th intercostal space on the anterior axillary line, as schematically shown in Fig. 7 [28]. Another interesting case series was published by Qu et al. on 3-arm biportal robotic sleeve lobectomies, with a similar approach, but accesses shifted upwards of one intercostal space [29]. The first uniportal RATS (uRATS) experience was published by Gonzales-Rivas et al., using the recent da Vinci SP model on cadavers [30]. This robotic platform is based on a single port, with an articulated elbow for instruments and camera, that enables additional manoeuvring space (but robotic staplers are still not available [31]). They performed lobectomies and thymectomies, using subcostal and subxiphoid accesses, respectively. For lobectomies, the 2.5 cm in diameter robotic trocar was positioned through a GelPOINT device (Applied Medical Corporation, Rancho Santa Margarita, California, USA), which allowed both CO_2_ delivery and positioning of an assistant port. From that point on, further runs were made with the da Vinci Xi model adopting a 3-arm approach. Performed procedures comprise lobectomy, segmentectomy, and sleeve lobectomy [32–36, 71]. The camera port is usually positioned in the most posterior part of the incision and instruments should work in parallel and never cross to reduce conflicts. Access location is variable in literature, ranging from 4 to 7th intercostal space, usually on the midaxillary line. Gonzales-Rivas et al. suggested the 7th intercostal space because lower access positioning can help the use of robotic staplers without limiting the procedure. A schematic representation is shown in Fig. 7 [72, 73]. Many authors stated that an experienced uniportal VATS surgeon is mandatory at the operating table [31, 33, 35]. Gonzales-Rivas et al. reported that one of the advantages is the fast undocking for conversion, and posterior segmentectomies are easily performed [31]. However, the learning curve is time-consuming. The authors recommended the use of robotic stapler to perform challenging steps without the assistant’s help.

### Outcomes of robotic lung cancer surgery

#### Surgical outcomes

A recent large meta-analysis based on 34 retrospective studies compared open surgery, VATS, and RATS lobectomies and revealed that mean difference of operative time was significantly higher in the RATS lobectomy group when compared to open surgery (mean difference = 61 min) and VATS (difference = 56 min) [37]. The risk ratio (RR) of the conversion rate was 0.55, thus not statistically significant when comparing VATS to RATS. Reoperation rate was lower in the VATS group, compared both to open surgery (RR = 0.51) and RATS groups (RR = 0.47). The mean difference of hospital length of stay was statistically significantly lower both in RATS (− 2.17 days) and VATS (− 1.86 days) groups when compared to open surgery. However, results should be carefully interpreted because RATS is a recent and developing technique. Given the absence of standardization, there may be some biases, as the one related to the learning curve.

An American multicentre, retrospective analysis was made on 5721 lobectomy cases, performed by open surgery, VATS, and RATS techniques [38]. Length of stay and chest tube duration were lower in the RATS group (*p* < 0.0001). Perioperative complications were similar across the minimally invasive approaches (*p* = 0.07), significantly lower than the open surgery approach (*p* < 0.0001 and *p* = 0.001). No difference in 30-day mortality was reported between groups (*p* not available). Conversion rate to open surgery with RATS was lower than with VATS (*p* not available). In contrast with other studies, operative time was lower in the RATS than in open surgery and VATS groups (the latter being the longest).

Notably, results by the ROMAN multicentre, randomized controlled trial which compared perioperative outcomes of RATS vs VATS lobectomy were published in 2021 [39]. The study was closed before complete accrual because periodic analysis showed no statistically significant difference in primary endpoints, such as rate of conversions, bleeding, and complications. RATS was superior to VATS in lymphadenectomy (total number of lymph nodes harvested *p* < 0.001 and station dissected *p* = 0.02) in terms of secondary outcomes. Nodal upstaging between groups was similar. Two more trials are ongoing. The first one has recently reported its preliminary results. In this single-centre open-label trial, RATS was superior to VATS in the primary endpoint, lymph node dissection (but not in nodal upstaging). [40]. The primary endpoint of the latter, a multicentre, randomized controlled trial, is health-related quality of life [41].

In a large RATS lobectomy series of 500 cases, Herrera et al. reported interesting results: an elective conversion rate of 5%, an emergency conversion rate of 0.6%, and a 30-day mortality of 0.6% [42]. Most frequent complications were atrial fibrillation (14.2% of cases) and prolonged air leak (9.8% of cases). The mean length of stay was 3.7 days (1–40).

In 2019, Cao and colleagues published a retrospective study reviewing the incidence and management of intraoperative catastrophes (defined as requiring emergency thoracotomy or a second unplanned major surgical operation) in robotic pulmonary resection [43]. In 1.9% of cases an intraoperative catastrophe occurred. The most common events were injuries to the pulmonary artery (80%) or pulmonary vein (6%). The most important referred factor for artery damage was the presence of hilar lymphadenopathy, with calcification or adhesion.

Even more recent techniques such as biportal and uniportal RATS have performed well with satisfactory operative outcomes. In particular, biportal demonstrated similar operative times to standard robotic approaches and conversion rate (0%) [28] and perioperative complications were satisfying [28, 29].

As stated above, extensive evidence is available for robotic lobectomy for lung cancer. However, a report on almost every procedure carried out with robotic systems has been published. Some results are briefly described in the following pages.

Some Authors state that RATS segmentectomy has the benefit of a higher camera definition, which makes it easier to recognize small vessels, and an integrated infrared light for indocyanine green that is helpful for intersegmental planes [44]. In an analysis of the National Cancer Database, Kodia et al. compared open surgery, VATS, and RATS segmentectomies [45]. Between the last two, perioperative outcomes were similar (e.g., conversion rate to open surgery, mortality, hospital length-of-stay) but a higher number of dissected lymph nodes (*p* < 0.01) were found for RATS, but surprisingly no nodal upstaging. To date, the two largest robotic segmentectomies series count 245 [46] and 121 [47] patients, with reported mean operative times of 86 and 114 min and conversion rates of 0.8 and 3.3%, respectively.

An important series of robotic sleeve lobectomies included 67 patients [48]. A subsequent article was published by the same centre, comparing operative outcomes between open surgery and VATS procedures [49]. Interestingly, surgeons opting for RATS performed better (i.e., operative time, blood loss, chest tube duration with *p* < 0.001). No conversions from RATS to open surgery were reported. Bronchial suturing time was similar between RATS (21–27 min), open surgery, and VATS groups; however, some surgeons argue that suturing is easier if performed by robot. A further analysis of oncological outcomes demonstrated important results, especially when compared to open surgery and VATS series from the literature [50].

Experience of lobectomy en bloc with chest wall has been reported to be feasible and safe, even if reported cases are scarce [51].

As far as pneumonectomy is concerned, the largest reported series counts 13 patients and analysed the conversion rate to open surgery (around 35% of cases), and operative outcomes, with similar results to VATS procedures [52]. Extraction of the specimen was feasible; however, it required a 4–5 cm incision. [53].

#### Oncological outcomes

To date, many retrospective articles have been published on the oncological adequacy of RATS lobectomy compared to both open surgery and VATS. Definitive data analysis on outcomes is still ongoing. However, preliminary results suggest equivalent radicality and survival. In addition, some series report even more successful lymphadenectomy with a higher number of resected lymph nodes and stations and an increased rate of nodal upstaging [37, 54, 55]. In fact, the previously cited meta-analysis reported that RATS achieved a higher number of resected lymph nodes (11.5) and stations (4.5) than VATS (10 and 3.5, respectively) but was comparable to open surgery in terms of oncological outcomes. Positive resection margins rate and 5-year overall survival were similar across treatments [37]. Other series on cN0 NSCLC analysed the frequency of nodal upstaging after RATS lobectomy drawing a comparison with other procedures. Zirafa et al. found a non-significant higher upstaging rate of 21% with RATS lobectomy compared to 18% of open surgery (*p* = 0.045) [56]. However, the difference was significant in cN0 to pN2 upstaging. Gallina et al. reported similar results, with a statistical significance in cN0 to pN2 nodal upstaging with RATS compared to VATS (11% vs 6%, *p* = 0.04) [57]. In the previously cited trials (both ROMAN and RVlob), no significant upstaging difference was found between RATS and VATS [39, 40], as well as in other retrospective series. In particular, Tang et al. compared open surgery (*n* = 50,186) and RATS (*n* = 7453) lobectomies for < cT2N0 NSCLC, based on National Cancer Database (NCDB) data, reporting a similar upstaging frequency (11.6% vs 11%, *p* = 0.28) but a higher number of resected lymph nodes for RATS (*p* < 0.001) [58]. In a different analysis stemming from the same database on open surgery (*n* = 13,725), VATS (*n* = 5112), and RATS (*n* = 1996) lobectomies for T3-T4 N0 NSCLC, Merritt et al. found similar upstaging incidence across procedures, both from N0 to N1 (11.9% vs 11.5% vs 11.8%, *p* = 0.274) and from N0 to N2 (6% vs 5.6% vs 5%, *p* = 0.274), but confirming a higher mean number of resected lymph nodes (*p* < 0.001) for RATS [59]. Notably, open lobectomies had a higher frequency of positive surgical margins (*p* < 0.001). A recent large series on RATS sleeve lobectomies for stage I-III NSCLC reported extremely satisfying 5-year overall survival (73%) and disease-free survival (67.9%) rates, especially when compared to literature data of VATS and open surgery [50].

## Discussion

Current evidence suggests that RATS is a valid minimally invasive technology, which proves not less than the pre-existing VATS. Its application in lung cancer treatment is gradually growing in popularity and the quality of several surgical gestures can improve through its employment. Lymphadenectomy proved to be accurate and safe; suturing, specifically during bronchial anastomosis in sleeve procedures and diaphragm plication, is effective and fluid [8, 9, 60]. Comparison with VATS is based only on retrospective studies, and definitive evidence is still lacking. However, many Authors reported positive experiences [8, 9]. The application of RATS in mediastinum and thymus surgery has been met with greater enthusiasm when compared to lung surgery. Manoeuvrability and view are higher in this complex and narrow anatomical region, which may theoretically lead to more extended and precise resections and less tissue damage. In particular, some Authors stated that the performance of the subxiphoid approach may be maximized using RATS [61]. However, the only clear evidence available at the present time is the superiority of RATS to trans-sternal thymectomy with reference to surgical outcomes [62, 63]. Few retrospective studies assessed the differences between RATS and VATS thymectomy. The only relevant result is the reduced hospital length of stay and blood loss for RATS; nevertheless, this result is not consistent across studies [64, 65]. Results regarding operative times are not straightforward either and may depend on different factors (e.g., previous VATS experience, centre volume) [64–66].

Several different RATS approaches are described for lung surgery, but a comparison study has not been published yet. Anyway, reported results for both portal and assisted robotic lobectomy approaches across studies are satisfying. The choice of a particular approach is probably dictated by the surgeon’s preference and experience, along with the experience of the centre. However, the surgeon should take advantage of the strength of either specific approach, depending on the encountered setting [74]. CO_2_ insufflation surely increases the operating field as it helps the visualization and the exposure of structures. However, if an RA approach is used, it may be time-consuming and less effective. With the advent of the da Vinci Xi many surgeons switched from a 3-arm to a 4-arm approach. Using all 4 arms favours the independence of the operator and reduces possible limitations of the assistant’s help.

Surgical and oncological outcomes data published so far suggest that RATS is a safe and effective technique. Some retrospective studies reported a decrease in hospital length of stay, perioperative complications, chest tube duration, and even operative times in some series, when compared to VATS [38, 67]. In some studies, conversion to open is less frequent in RATS than in VATS [38, 75], but results are not consistent across literature [37, 39, 45]. Nevertheless, conversion rate remains < 10% in recent series, thus at least not higher than VATS [37]. Reasons for conversion are similar to those described for VATS (e.g., uncontrollable bleeding, extensive adhesions, anatomical reasons, and lymph nodes of complex resection) [38, 42, 75–77]. It should be noted that there are several procedures in which robotic systems may even perform better than VATS. Interest in robotic sleeve lobectomies has grown and some Authors reported a better suturing experience with RATS [8, 9, 50, 60]. Related outcomes seem satisfying in retrospective series, even when compared to VATS and open surgery [50]. Moreover, RATS generally achieves a better lymphadenectomy in several studies, ensuring higher radicality and more precise disease staging [37, 39, 40, 54, 55]. As a fact, a missed N disease is at higher risk of disease recurrence. Thus, RATS might ensure a higher oncological radicality than VATS. However, evidence regarding nodal upstaging frequency is still not straightforward [37, 39, 57].

Still, the most diving issue about RATS is the benefit/cost balance. A careful assessment should be determining in which procedure RATS achieves better results than other approaches with affordable expense. Generally, if robotic surgery could reduce complications rate and length of hospitalization, even with initial higher expenses, the benefit would outweigh the initially higher costs. However, more efforts should be done in the direction of lowering costs to facilitate the wide introduction of this valid technology. It is likely for the spread of RATS and the commercialization of new robotic platforms to help to reduce prices.

Some RATS disadvantages could be potentially compensated during the learning curve, such as the absence of haptic feedback, that may be replaced by the high-definition 3D-view stable optical system. In the future, the introduction of tactile feedback replacing technologies may further reduce this limit. Operative times may be importantly reduced with experience and practice, as demonstrated in some series. Many studies report that docking times may already decrease after 15–20 procedures [68]. The learning curve should not be viewed as an obstacle. VATS learning curve seem to be tougher than RATS (30/50 vs 20 lobectomies) [69]. A single surgeon experience on 150 procedures study suggested a faster reduction in operative time for RATS than for VATS (32 vs 34 lobectomies), in contrast with a slower reduction of surgical failure (32 vs 28) [70]. However, current literature is still lacking a precise and complete analysis of the learning curve difference between the two techniques. More evidence is needed. RATS comparison with open surgery brings interesting results. The winning idea behind robotic surgery is to perform surgery as precisely as open surgery but being as mini-invasive as VATS at the same time. If it demonstrates easier than VATS, and its outcomes equal or superior to open surgery, along with a reduction of costs, it could be the mind-changer technology for open surgeons where VATS failed. In addition, the development of uniportal and biportal RATS approaches may further accommodate open surgeons to approach minimally invasive surgery.

The main limitation of the present narrative review is its non-systematic nature; nevertheless, it offers an updated overview on the main RATS lobectomy approaches, as well as the main evidence available on operative and oncological outcomes of RATS for lung resections.

## Conclusions

Robotic thoracic surgery for lung cancer has proven to be a safe and effective technique. Literature is considerable and rapidly expanding, and more evidence will be available in the future. However, results on surgical and oncological outcomes are, if not better than, at least comparable to those obtained by VATS. We believe that RATS could improve outcomes in determined settings, such as sleeve lobectomies. Several different RATS lobectomy approaches are described, and the choice should be based on the experience and the needs of the surgeon and the team. Moreover, the emerging biportal and uniportal RATS appeared feasible and safe, and more evidence is looked ahead to. Ideally, one should not focus all their attention on just one approach or technique during surgery. The most fascinating aspect is the fact that different solutions are available for the same problem; therefore, advantages and disadvantages must be outweighed according to each specific setting. Consequently, robotic systems should not be viewed as the only valid solution to thoracic surgery problems but as an additional minimally invasive option. Experience to identify the best candidates and the optimal procedures to be applied to is required. As a tool, it can minimize all hand-related defects (e.g., tremors, involuntary movements, inaccuracy), guaranteeing more hand-factor homogeneity across surgeons. The surgeon’s indication remains crucial.

## Data Availability

Data available upon request.
